# Lysine Acetylome Study of Human Hepatocellular Carcinoma Tissues for Biomarkers and Therapeutic Targets Discovery

**DOI:** 10.3389/fgene.2020.572663

**Published:** 2020-09-17

**Authors:** Qianwei Zhao, Zhendong Zhang, Jinxia Li, Fang Xu, Bingxia Zhang, Mengduan Liu, Yixian Liu, Huiping Chen, Junxia Yang, Jintao Zhang

**Affiliations:** ^1^BGI College and Henan Institute of Medical and Pharmaceutical Sciences, Zhengzhou University, Zhengzhou, China; ^2^Henan Key Laboratory for Pharmacology of Liver Diseases, Zhengzhou University, Zhengzhou, China; ^3^School of Basic Medical Sciences, Zhengzhou University, Zhengzhou, China; ^4^School of Life Sciences, Zhengzhou University, Zhengzhou, China

**Keywords:** hepatocellular carcinoma, lysine acetylation, non-histone proteins, sirtuins, biomarker candidates, drug discovery

## Abstract

Lysine acetylation is a vital post-translational modification (PTM) of proteins, which plays an important role in cancer development. In healthy human liver tissues, multiple non-histone proteins were identified with acetylation modification, however, the role of acetylated proteins in hepatocellular carcinoma (HCC) development remains largely unknown. Here we performed a quantitative acetylome study of tumor and normal liver tissues from HCC patients. Overall, 598 lysine acetylation sites in 325 proteins were quantified, and almost 59% of their acetylation levels were significantly changed. The differentially acetylated proteins mainly consisted of non-histone proteins located in mitochondria and cytoplasm, which accounted for 42% and 24%, respectively. Bioinformatics analysis showed that differentially acetylated proteins were enriched in metabolism, oxidative stress, and signal transduction processes. In tumor tissues, 278 lysine sites in 189 proteins showed decreased acetylation levels, which occupied 98% of differentially acetylated proteins. Moreover, we collected twenty pairs of tumor and normal liver tissues from HCC male patients, and found that expression levels of SIRT1 (*p* = 0.002), SIRT2 (*p* = 0.01), and SIRT4 (*p* = 0.045) were significantly up-regulated in tumor tissues. Over-expression of possibly accounted for the widespread deacetylation of non-histone proteins identified in HCC tumor tissues, which could serve as promising predictors of HCC. Taken together, our work illustrates abundant differentially acetylated proteins in HCC tumor tissues, and offered insights into the role of lysine acetylation in HCC development. It provided potential biomarker and drug target candidates for clinical HCC diagnosis and treatment.

## Introduction

HCC is one of the most common human cancer types and accounts for about 90% of primary liver cancer (El-Serag and Rudolph, 2007). Because of its high degree of malignancy, recurrence, metastases, and poor prognosis, HCC is the second deadliest human cancer in the world. The 5-year survival rate of HCC patients at advanced stage is less than 10% while patients at early stage can experience significantly improved survival (Hiwatashi et al., 2015). To explore effective biomarkers for HCC diagnosis at early stage, multiple kinds of omics studies have been performed with samples from HCC patients or HCC cell lines, such as genomics, transcriptomics, proteomics, metabolomics, and so on. These omics studies provided huge number of molecules including circulating tumor DNA (ctDNA), circulating miRNAs, proteins, and metabolites as biomarker candidates for clinical HCC diagnosis (Tang et al., 2016; Zhou et al., 2011; Dittharot et al., 2018; Gao et al., 2019). Based on the data from omics studies, several effective biomarkers were identified and validated in clinical samples from HCC patients, among which AFP was the most widely used protein biomarker for early diagnosis of HCC (Marrero et al., 2009). However, clinical studies showed that the sensitivity and specificity of one protein marker was limited in detecting HCC at early stage (Sun et al., 2008).

In recent years, with the development of biotechnology and its application in omics study, different types of protein PTM were identified and researches indicated that protein PTM was closely associated with disease development. Protein PTM was usually evolutionarily conserved and regulated protein stability, localization, function, protein-protein interaction, and protein-nucleic acid interaction. Omics studies of protein PTM including ubiquitination, methylation, glycosylation, acetylation, crotonylation, and lactylation showed significant difference between tumor and normal samples, which indicated that protein PTM played an important role in cancer development (Zhang et al., 2019; Wan et al., 2019; Zhong and Huang, 2016; Sun et al., 2006; Chen et al., 2018). For example, studies showed that the glycosylation levels of proteins significantly changed in different types of tumor tissues, and some glycosylated proteins were promising biomarkers for diagnosis of HCC at early stage (Wang et al., 2017; Block et al., 2005). Therefore, omics study of protein PTM was of great importance because it not only provided new insight into mechanism study of proteins in cancer development, but also served as new candidates for biomarker and therapy target discovery.

Lysine acetylation was a kind of protein PTM that is involved in various cellular processes, such as metabolic pathways, signal transduction, cell proliferation, migration and apoptosis. Increasing evidence showed that lysine acetylation participated in development of multiple kinds of cancers (Martile et al., 2016; Gil et al., 2017). For example, Lysine acetylation on histones could change local chromatin structure for transcription factors to bind and initiate gene transcription (Jin et al., 2011). Lysine acetylation on non-histone proteins including enzymes and transcription factors induced metabolic rewiring and gene transcription, and affected cell proliferation, apoptosis, and metastasis (Leo et al., 2019; Thangjam et al., 2016; Wang B. et al., 2020; He et al., 2019). These findings indicated that lysine acetylation was an important factor in cancer development. In human liver tissues, more than 1000 acetylation sites in proteins were identified, among which metabolic enzymes accounted for a large amount (Zhao et al., 2010). Increasing evidence showed that lysine acetylation modification on enzymes played a vital role in metabolic processes during liver disease development (Weems and Olson, 2011; Cao et al., 2017; Hu et al., 2017; Gu et al., 2020). Besides, most members of histone acetylase (HAT) and histone deacetylase (HDAC) families were reported to be aberrantly expressed in HCC tumor tissues, which were associated with clinical stage, prognosis, and survival rate, and some HDACs inhibitors were taken as candidates for clinical HCC treatment (Li et al., 2011; Quint et al., 2011; Freese et al., 2019; Hu et al., 2019; Inagaki et al., 2016). Taken together, it showed that lysine acetylation was playing an important role in HCC initiation and development. Therefore, it is necessary to study lysine acetylation in liver tissues of HCC patients thoroughly. Here, we performed lysine acetylome study of HCC tumor and normal liver tissues, and identified multiple proteins with differential acetylation levels. The differentially acetylated proteins mainly consisted of non-histone proteins located in mitochondria and cytoplasm. Besides, SIRT1, 2, and 4 showed increased expression levels in tumor tissues, which probably accounted for widespread deacetylation of the non-histone proteins, and may serve as diagnostic predictors of HCC.

## Materials and Methods

### Experimental Design and Statistical Rationale

The tissue samples were selected from HCC male patients (stage II) with an average age of 43 (41–47), who had not been treated before. The para-carcinoma normal liver tissues around the tumor tissues from the same HCC patients were selected as control samples. Principal Component Analysis (PCA) method was used to represent the correlation between tumor and normal liver tissues (Supplementary Figure S1). The acetylation intensity of tissue samples was quantified by four full-quantitative quantitative experiments. First step, we calculated differential abundance of the acetylation between the cancer and normal tissue samples. In brief, we firstly calculated the average value of the quantitative values of each sample in four biological replicates, and then we calculated the ratio of the average values between the cancer and normal samples. The ratio was taken as the final quantitation. Next step, the significant *p* value of differential expression between two samples was calculated. The relative quantitative values of each sample were taken as log2 transform (so that the data conforms to the normal distribution), and *p* value was calculated by the two-sample two-tailed *T*-test method. Among the differentially expressed proteins (with a corrected *p* value < 0.05), it was considered as significant up-regulation or down-regulation when the fold change was greater than 1.5 or less than 0.67. Raw abundance ratios of acetylation sites were normalized based on the corresponding protein ratio. Negative logarithm (-log10) transformation was carried out for *p* value (obtained from Fisher’s exact test) to indicate the significance of GO, KEGG, and protein domain enrichment.

### Sample Preparation

Five pairs of tumor and para-carcinoma normal liver tissues from HCC patients were surgically resected in the First Affiliated Hospital of Zhengzhou University, with approval from the Research Ethics Committee of Zhengzhou University and consent from the patients. The tumor tissues were washed in PBS and surrounding tissues were removed. The para-carcinoma normal liver tissues were managed with the same method. The whole process took place on ice, and it was finished within 30 min after the surgery. The collected tissues were divided into small pieces and stored in 1.5 ml tubes, which were kept in liquid nitrogen. The selected samples were renamed with codes (A1, A2, C1, C2, D1, D2, E1, E2, F1, F2, the number 1 represented tumor tissues while 2 represented normal tissues) instead of the patient′s name in this study.

### Protein Extraction and Digestion

Samples were homogenized by a high intensity ultrasonic processor (Scientz, Germany) in 1 mL lysis buffer (8 M urea, 3 μM TSA, 50 mM NAM, 2 mM EDTA, and 1% Protease Inhibitor Cocktail). Then the lysate was centrifuged at 12000 g for 10 min at 4°C, and the supernatant was transferred to new tubes. The concentration of the protein was determined by BCA Protein Assay Kit (Sangon Biotech, China) before use. For digestion, the protein was first reduced with DTT (5 mM) for 30 min at 56°C. Then iodoacetamide was added to the protein to make its final concentration of 11 mM, and the mixture was incubated for 15 min at room temperature in the dark. Finally, the concentration of urea in the protein solution was diluted to less than 2 M by adding triethyl ammonium bicarbonate (TEAB, 100 mM). The protein was digested by trypsin with a trypsin/protein mass ratio of 1/50 at 37°C overnight, and then additional trypsin was added to the protein with a trypsin/protein mass ratio of 1/100 and incubated at 37°C for 4 h.

### TMT Labeling and Affinity Enrichment of Acetylated Peptides

Tryptic peptides were freeze-dried after being desalted by using Strata X C18 (Phenomenex, United States). Then the peptides were dissolved in TEAB (0.5 M) to be labeled by TMT tag according to the protocol of the TMT labeling kit (Thermo, United States). TMT reagents were thawed and reconstituted in acetonitrile. The peptide mixtures were incubated for 2 h at room temperature, and then desalted and dried by vacuum centrifugation. Next, TMT labeled peptides were dissolved in NETN buffer (100 mM NaCl, 1 mM EDTA, 50 mM Tris–HCl, 0.5% NP-40, pH = 8.0), and the supernatant was collected and incubated with agarose beads coupled to anti-acetyl antibody PTM-104 (PTM Bio, Jingjie, China) overnight at 4°C with gentle shaking. After, the beads were washed with NETN buffer four times, and then twice with ddH_2_O. Finally, the bound peptides were eluted with 0.1% trifluoroacetic acid. The eluent was vacuum-dried and then desalted by C18 ZipTips (Millipore, United States) according to the manufacturer’s instruction. The TMT labeled peptides were freeze-dried for Liquid chromatography-tandem mass spectrometry (LC-MS/MS) analysis.

### Protein Identification and Quantification by LC-MS/MS Analysis

LC-MS/MS analysis was performed according to the literature procedure. The EASY-nLC 1000 UPLC system was used to separate peptides dissolved in 0.1% formic acid (FA). The mobile phase consisted of buffer A (0.1% FA and 2% acetonitrile in ddH_2_O) and buffer B (0.1% FA and 90% acetonitrile in ddH_2_O); it was eluted with a linear gradient of eluent, starting with 10% buffer B which was increased to 25% in 40 min, and then from 25% to 38% for 12 min, and later from 38% to 80% for 4 min, finally, it was kept at 80% buffer B for 4 min. The flow rate was 700 nL/min. The peptides were subjected to the NIS ion source and then performed MS/MS analysis by Orbitrap Fusion^TM^ (Thermo, United States) system. The electrospray voltage applied was 2.0 kV. For MS1 scan, it was set at a resolution of 60,000 with a scan ranging from 350 to 1550 m/z. For MS2 scan, it was set at a resolution of 30,000 (100 m/z). Data were generated by using the Data-Dependent Acquisition (DDA) strategy.

### Database Search

The MS/MS data was performed by Maxquant search engine (v1.5.2.8), and tandem mass spectra was searched against SwissProt Human (20130 sequences) database. FurtheMore, additional libraries were added for false discovery rate (FDR) calculation and elimination of contaminated protein′s influence. Trypsin/P was specified as the cleavage enzyme allowing up to 4 missing cleavages, 7 modifications per peptide and 5 charges. Mass error was set to 10 ppm and 5 ppm for precursor ions of first and main searches, respectively, and mass error for fragment ions was set to 0.02 Da. Carbamidomethyl on Cys was specified as fix modification, while oxidation on Met and acetylation on Lys and N-terminal of protein were specified as variable modifications. Quantitative method was set to TMT-10plex. FDR thresholds for protein, peptide and modification sites were specified at 1%.

### Bioinformatics Analysis

Gene Ontology (GO) annotation proteome was performed from the UniProt-GOA database^1^, and proteins were classified into three categories: biological process, cellular component and molecular function. Kyoto Encyclopedia of Genes and Genomes (KEGG) pathway analysis was based on database^2^. Subcellular location was derived from the software named WoLF PSORT. Protein domain was annotated by soft InterProScan based on InterPro domain database^3^. Motif-x software was employed to analyze the model of sequences constituted with amino acids in specific positions of acetyl-21-mers (10 amino acids upstream and downstream of the site) in all the protein sequences. Protein-protein interaction (PPI) network analysis was performed by STRING 10.5^4^.

### Western Blot Analysis

About 100 mg tumor or normal liver tissues were acquired and digested in 1 mL RIPA lysis buffer on ice for 30 min. The lysate was centrifuged for 20 min, 12000 rpm at 4°C, protein extraction samples were collected and concentration were detected with a microplate reader. Samples were boiled at 95°C for 5 min and directly loaded onto a 10% SDS-PAGE gel. Then proteins were transferred to a PVDF membrane. The membrane was incubated within 5% non-fat dry milk in TBS with 0.1% Twee-20 (TBS-T) to block the protein spots, and then it was incubated with primary antibodies (SIRT1, 2, and 4 from BBI, China) at 4°C overnight. The membrane was washed with TBS-T for three times and incubated in secondary antibody. Signals were detected with the Amersham Imager 600 System (General Electric Company, United States). GAPDH was used as internal control. The expression levels were quantified by measuring the intensity of each band with Image J software.

## Results

### Identification and Analysis of Acetylated Proteins in HCC Tumor and Normal Liver Tissues

**FIGURE 1:**
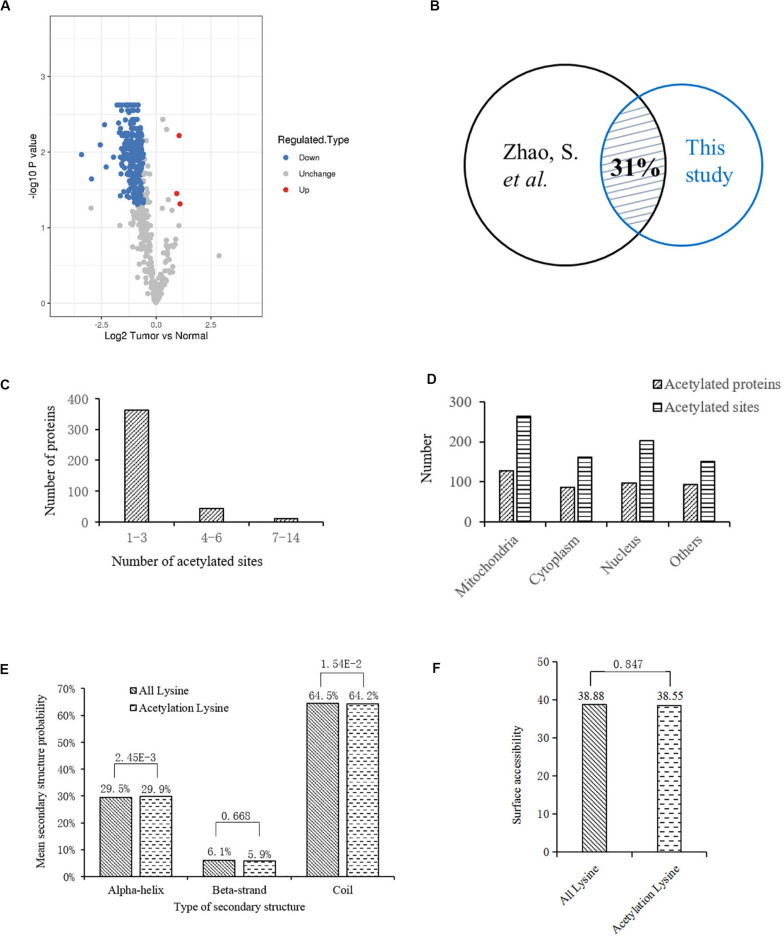
Bioinformatics analysis of lysine acetylation sites identified in liver tissues of HCC patients. **(A)** Volcano plot showing the distribution of acetylation sites rations (Tumor vs. Normal). **(B)** Comparison of two acetylation proteomic studies in human liver tissues: this study and reference (Zhao et al., 2010). **(C)** Statistic analysis of acetylated sites in the acetylated proteins. **(D)** Distribution of acetylated sites and proteins in different cellular components. **(E)** Distribution of lysine acetylation sites in protein secondary structures. **(F)** Predicted surface accessibility of acetylation sites.

To profile the protein lysine acetylation of liver tissues during HCC development, five pairs of primary tumor and para-carcinoma normal liver tissues were surgically resected from HCC patients. Four pairs of qualified tissues were selected for further analysis after quality test. Proteins were extracted from the collected liver tissues and then digested into peptides by trypsin. After being labeled with TMT tag, the digested peptides with lysine acetylation sites were enriched and analyzed by LC-MS/MS. Based on the human Uniprot database, we identified 792 acetylation sites in 415 proteins, among which 598 acetylation sites in 325 proteins were quantified. Compared to the normal liver tissues, 278 acetylation sites in 189 proteins were down-regulated while three acetylation sites in three proteins (EP300, GRHPR, and GLDC) were up-regulated with significant differences in tumor tissues (Supplementary Table S1; Figure 1A). In a previous acetylation proteomics study, more than 1000 acetylated proteins were identified in human liver tissues (Zhao et al., 2010). By comparison of these two studies, we found that about 31% of proteins with differential acetylation levels identified in our study were present in the previous study (Figure 1B). It indicated that the spectrum of acetylated proteins was highly conserved in human liver tissues, and the proteins with aberrant acetylation levels in tumor tissues probably participated in HCC development.

Then we investigated the distribution of identified lysine acetylation sites in proteins by calculating the number of acetylation sites per protein. As shown in Figure 1C, most of the proteins contained no more than three lysine acetylation sites, which accounted for 87%, while 13% of them contained three more sites. Next, distribution of acetylated lysine sites and proteins were analyzed (Figure 1D), it showed that acetylated proteins were mainly associated with mitochondria (31%), nucleus (24%), and cytoplasm (22%). Besides, among the remaining 23% proteins, most of them were located in organelles including endoplasmic reticulum and peroxisome. To explore the relationship between lysine acetylation sites and protein secondary structures, a structure analysis of the acetylated lysine sites and all lysine sites was performed. As shown in Figure 1E, the α-helix and coil probabilities of acetylated lysine sites were significantly different from that of all lysine sites, while there was no obvious difference in β-strand probabilities between the acetylated lysine sites and all lysine sites. Additionally, surface accessibility of lysine acetylation sites analysis showed that about 38.55% of acetylated lysine sites were exposed to protein surfaces, compared to 38.88% of all lysine sites (Figure 1F). Therefore, lysine acetylation seemed to have little effect on surface property of identified proteins in HCC liver tissues.

**FIGURE 2:**
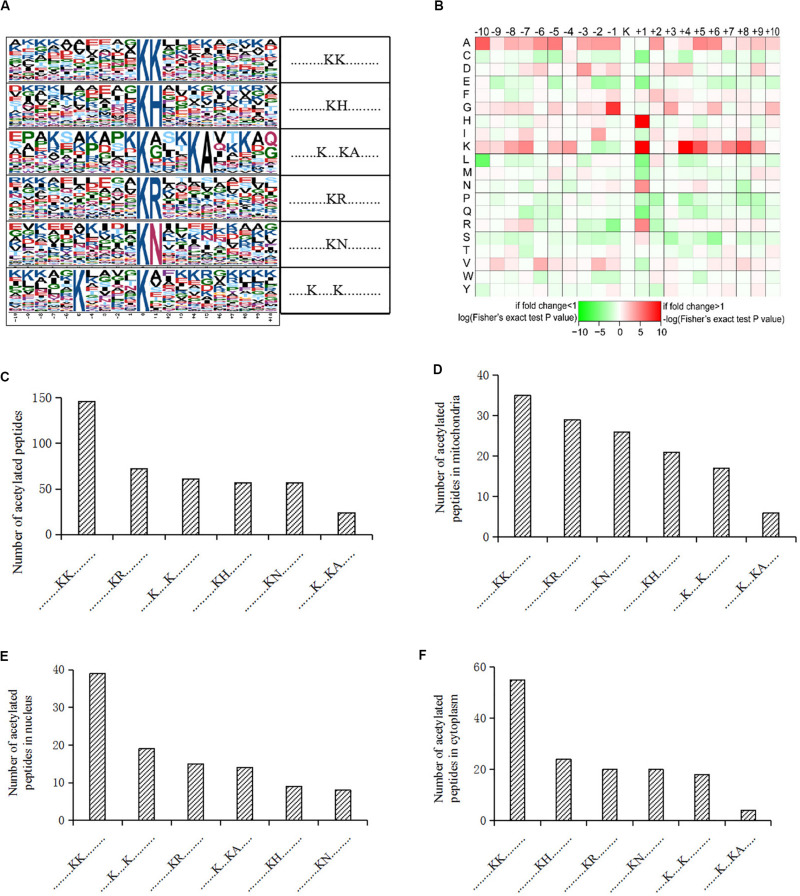
Motif analysis of acetylated peptides. **(A)** Acetylation motifs and amino acids surrounding the lysine acetylation sites. **(B)** Heat map representing the amino acids composition of the lysine acetylation sites. **(C)** Distribution of motifs in acetylated peptides. **(D–F)** Distribution of motifs in acetylated peptides associated with different cellular compartments.

We also analyzed the amino acids from the -10 to + 10 positions surrounding the identified lysine acetylation sites and searched for occurrences of amino acid motifs. Of all the acetylated peptides, 417 were matched to six conserved motifs, including KK.,.KH., ……….K…KA………., ……….KR………., ……….KN., and K. K (K indicates the acetylated lysine, and H, A, R, N represent histidine, alanine, arginine, and asparagine, respectively) (Figure 2A). In Figure 2B, local sequence context around the lysine acetylation sites was analyzed and it indicated that positively charged amino acids (K, H, and R) were almost completely excluded from the -1 position, while they were enriched in the + 1 position. This data suggested that according to the heat map of the amino acid compositions surrounding the acetylation sites, the frequency of G (G indicates glycine) in position -1 was the highest, and the frequency of K and A from the -10 to + 10 positions was mainly kept at a higher level in the motifs. Acetylated peptides containing motif. KK. accounted for 35% of all the identified peptides, while peptides containing motifs. KR.,.K.K., ……….KH………., ……….KN………., and …….K.KA. accounted for 17.3%, 14.6%, 13.7%, 13.7%, and 5.8%, respectively (Figure 2C). In addition, the distribution of different motifs in cellular compartments were assessed, and results showed that acetylated peptides were predominantly associated with cytoplasm and mitochondria, where the motifs were similar, but different from nuclear motifs (Figures 2D–F).

### Functional Enrichment and Subcellular Location of Differentially Acetylated Proteins

**FIGURE 3:**
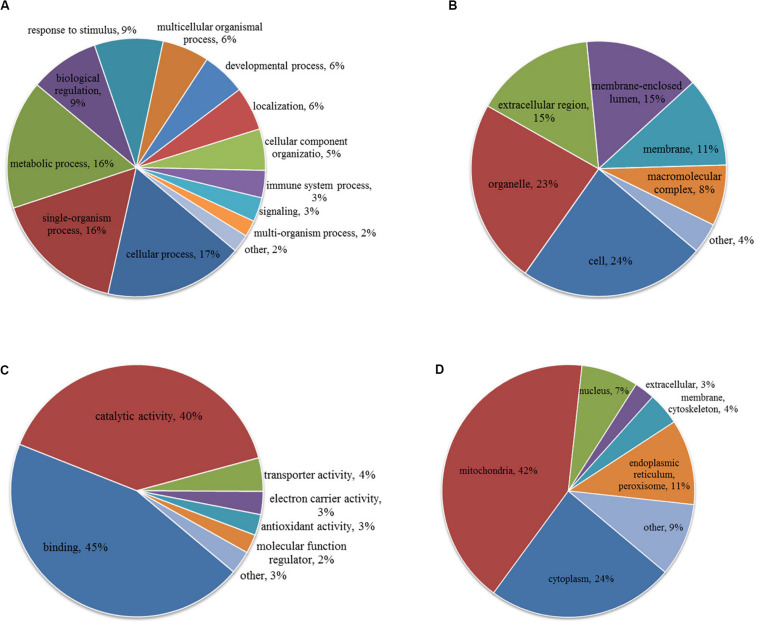
GO **(A–C)** and subcellular localization **(D)** analysis of differentially acetylated proteins. **(A)** Biological process. **(B)** Cellular component. **(C)** Molecular function.

To characterize the role of lysine acetylome in HCC development, we performed GO analysis on the differentially acetylated proteins based on biological process, cellular component, and molecular function (Figures 3A–C). Biological processes analysis indicated that acetylated proteins were enriched in cellular process (17%), single-organism process (16%), metabolic process (16%), biological regulation (9%) and response to stimulus (9%). Besides, according to cellular component analysis, there were 24% and 23% acetylated proteins located in cell and organelle, respectively, 15% in membrane-enclosed lumen, 15% in extracellular region, 11% in membrane, and 8% in macromolecular complex. Molecular function analysis showed that 45% and 40% acetylated proteins were involved in binding and catalytic activities, respectively, which accounted for most of the acetylated proteins. Taken together, GO analysis revealed that differentially acetylated proteins were enriched in metabolic, catabolic, and signal transduction processes. Consistently, subcellular location showed that differentially acetylated proteins were primarily associated with mitochondria and cytoplasm, which accounted for 42% and 24%, respectively. Less acetylated proteins were in located in endoplasmic reticulum and peroxisome (11%), nucleus (7%), membrane and cytoskeleton (4%), and extracellular (3%) (Figure 3D).

**FIGURE 4:**
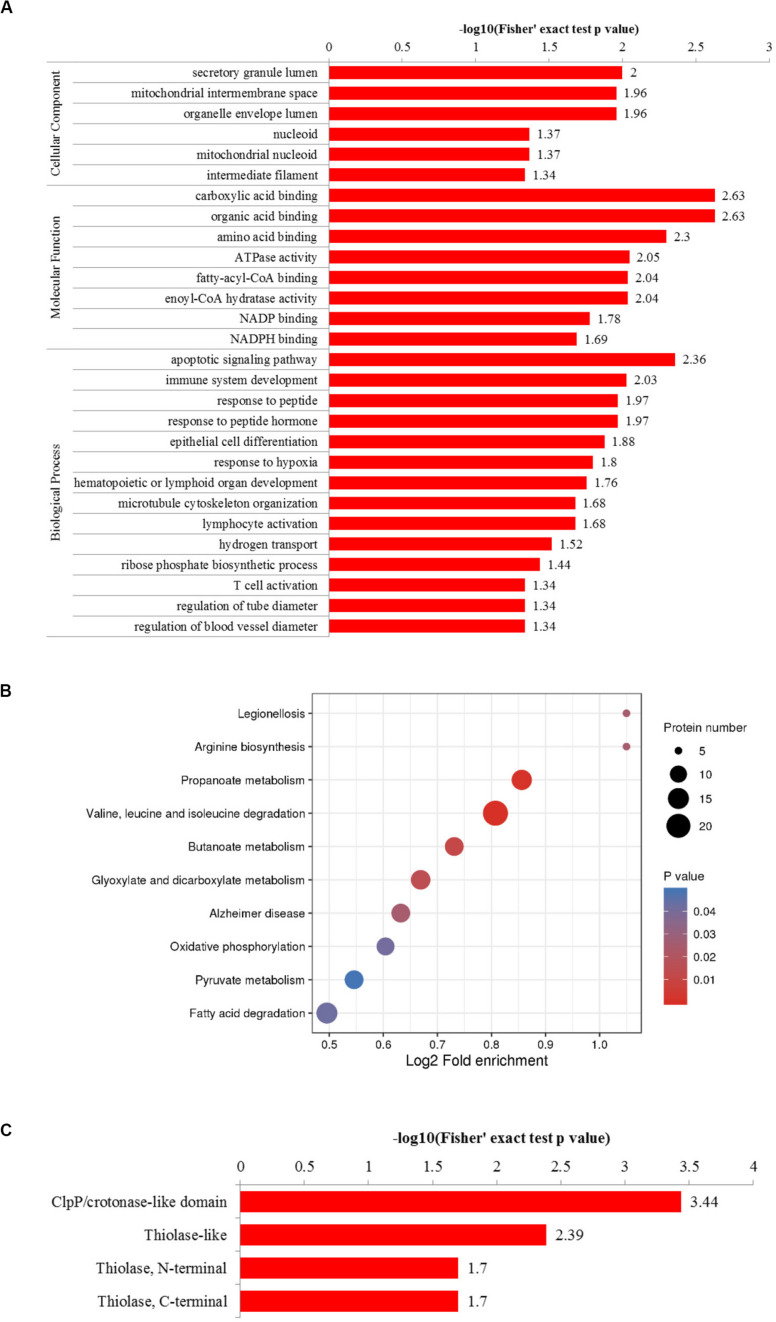
GO enrichment **(A)**, KEGG pathway enrichment **(B)**, and protein domain enrichment **(C)** analysis of differentially acetylated proteins.

**FIGURE 5:**
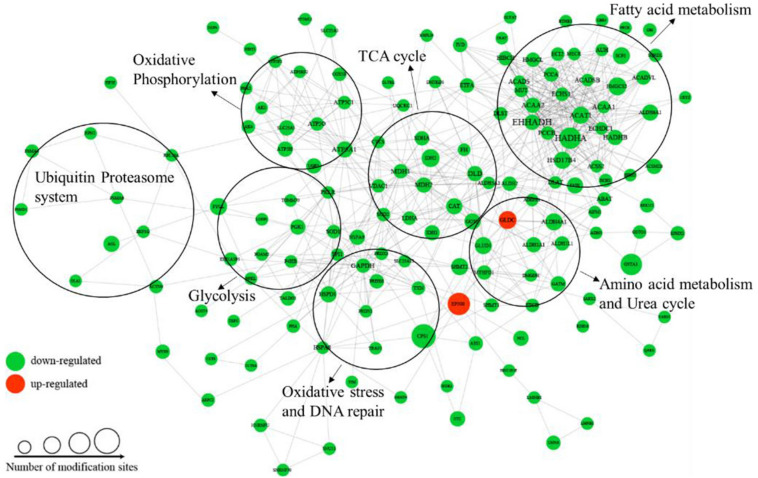
PPI network analysis of differentially acetylated proteins in HCC tumor tissues.

To better understand the cellular processes regulated by differentially acetylated proteins in HCC tumor tissues, GO and KEGG pathway enrichment analysis were carried out. As shown in Figure 4A, it showed that differentially acetylated proteins were markedly involved in epithelium development, mitochondria function, and intracellular metabolic processes. In agreement with this finding, KEGG pathway enrichment analysis demonstrated that differentially acetylated proteins participating in glucose, fatty acid, and amino acids metabolism pathways were mostly enriched (Figure 4B). Besides, protein domain enrichment analysis showed that CIpP/crotonase-like, Thiolase-like, Thiolase, N-terminal, and Thiolase, C-terminal domains were mainly enriched (Figure 4C). All these domains were involved in enzymatic activities, especially lipid metabolism. Additionally, the differentially acetylated proteins were subjected to PPI network analysis based on the STRING database, and it showed that proteins were clustered into three categories including metabolism, oxidative stress and DNA repair, and the ubiquitin proteasome system (UPS) (Figure 5).

### Disorder of Lysine Acetylation Modification on Metabolic Enzymes in HCC Tumor Tissues

**FIGURE 6:**
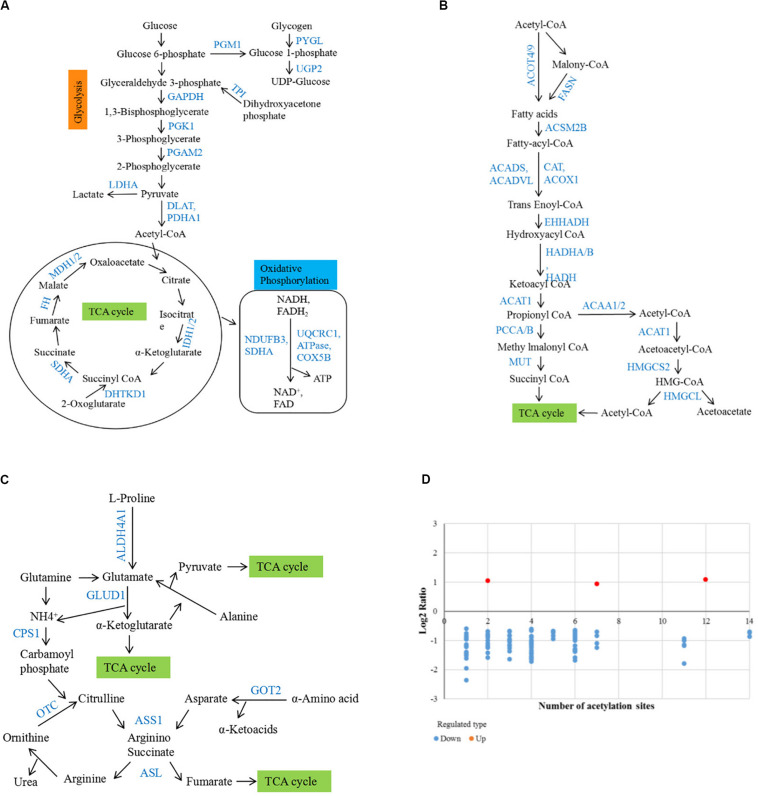
Involvement of differentially acetylated proteins in metabolic pathways. **(A–C)** Schematic representation of differentially acetylated enzymes (shown in blue) in glucose, fatty acid, and amino acid metabolism. **(D)** Distribution of lysine acetylation sites rations in metabolic enzymes (Tumor vs. Normal).

Among the differentially acetylated proteins identified in this research, metabolic enzymes represented a significant proportion, and they mainly participated in processes such as glycolysis, gluconeogenesis, the TCA cycle, fatty acid oxidation, glutamine metabolism, and the urea cycle (Figure 5). According to the statistical analysis, proteins participating in glucose, fatty acid and amino acid metabolism accounted for 46%, 39%, and 15%, respectively. In the glucose metabolic process, most differentially acetylated enzymes directly participated in glycolysis (GAPDH, PGK1, PGAM2, LDHA, DLAT, PDHA1), TCA cycle (IDH1/2, DHTKD1, SDHA, FH, MDH1/2), and oxidative phosphorylation (NDUFB3, SDHA, UQCRC1, ATPase, COX5B) processes (Figure 6A). Additionally, in fatty acid metabolic process (Figure 6B), about 60% of enzymes contained more than one lysine sites with differential acetylation levels. For example, we identified five lysine acetylation sites in HADHA, four lysine acetylation sites in HADHB, and three lysine acetylation sites in both ACADVL and PCCA, all of which showed lower acetylation levels in tumor tissues. Besides, several acetylated proteins with decreased lysine acetylation levels were located in peroxisome, where the main function was to catalyze the oxidation of fatty acid. Moreover, several pivotal enzymes involved in amino acid metabolism process and urea cycle were identified with decreased lysine acetylation levels in HCC tumor tissues, such as GLUD1, ASL, GOT2 and so on (Figure 6C). GLUD1 plays an important role in glutamine consumption, which is a common feature of HCC liver tissues and provides energy for tumor growth (Dang, 2013). Here we identified five lysine acetylation sites in GLUD1, and all the acetylation levels were down-regulated in tumor tissues. In general, most of the acetylation levels of lysine sites in enzymes decreased in tumor tissues (Figure 6D). Additionally, some of the lysine-acetylated peptides were confirmed by MS/MS spectra, with their relative intensities in HCC tumor and normal liver tissues (Supplementary Figures 2A–C).

### Involvement of Acetylated Proteins in Signal Transduction Process and Oxidative Stress in HCC Tumor Tissues

**FIGURE 7:**
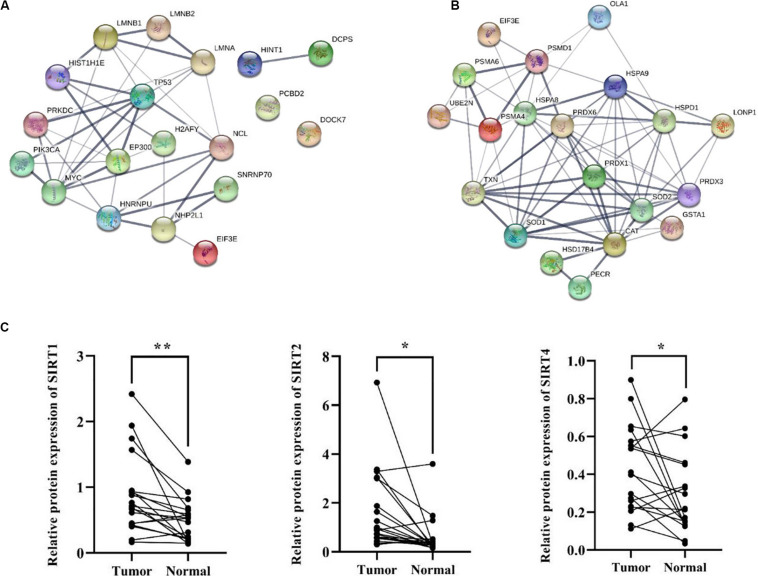
PPI network analysis of differentially acetylated nuclear factors **(A)** and oxidative stress associated proteins **(B)** in HCC tumor tissues. **(C)** Protein expression levels of SIRT1, 2, and 4 in tumor and normal liver tissues from HCC patients. *p < 0.05 and **p < 0.01 indicate significant differences.

Besides metabolic enzymes, transcription factors accounted for another large part of non-histone substrates of HATs and HDACs. Transcription factors usually function on genomic DNA and play important roles in genes transcription. In this research, nearly 10% of differentially acetylated proteins were located in nuclear, and most of them were involved in gene transcription process, such as chromatin organization, telomere dynamics, DNA/RNA binding, and mRNA splicing (Supplementary Figure S3). To evaluate the importance of these acetylated nuclear factors during HCC progression, here we performed PPI network analysis. Results in Figure 7A showed that most of the acetylated nuclear factors had direct and close interaction with important factors in HCC signal pathways, such as p53, PI3K, and c-Myc. Among the nuclear factors, EP300 is a transcriptional co-activator of which the K1550 site showed increased acetylation level in tumor tissues. Besides, as a member of HATs, EP300 had direct association with multiple non-histone proteins, among which several proteins identified in this study were included, such as HSD17B4, EHHADH, LDHA, SLC25A5, NCL, SMAD4, HSPA8, and so on.

HCC development usually accompanies oxidative stress, and antioxidant stress system deficiency is a common feature in tumor cells. In this study, most proteins involved in reactive oxygen species (ROS) and oxidative stress contained lysine acetylation sites, such as SOD1, SOD2, PRDX1, PRDX3, PRDX6, HSD17B4, PECR, GSTA1, TXN, and CAT. Compared to the normal liver tissues, the acetylation levels of lysine sites in these proteins were generally down-regulated in tumor tissues. As a response to oxidative stress and ROS, the damaged proteins are mainly degraded by the UPS, which is to protect cells from ROS damage (Marques et al., 2006). Consistently, several lysine sites of proteins involved in UPS showed decreased acetylation levels in tumor tissues (**Supplementary Table 2**). Moreover, heat shock proteins (HSPs) including HSPA8, HSPA9, and HSPD1 in tumor tissues displayed aberrant lysine acetylation modification. HSPs are molecular chaperones that play key roles in refolding denatured proteins. HSPs promoted tumorigenesis, tumor growth, and metastasis, and blockade of HSPs and activation of UPS induced protein degradation and prevented tumor development (Calderwood, 2018). Finally, twenty acetylated proteins were analyzed in a PPI network (Figure 7B), and it showed close interaction among proteins associated with oxidative stress in HCC tumor tissues.

### SIRT1, 2 and 4 Were Up-Regulated in HCC Tumor Tissues

According to the bioinformatics analysis, differentially acetylated proteins mainly consisted of non-histone proteins located in mitochondria and cytoplasm, most of which displayed decreased lysine acetylation levels in tumor tissues. Among HDACs, SIRT1-5 accounted for deacetylation of non-histone proteins and several acetylated proteins identified in tumor tissues were substrates of SIRT1-5. Therefore, we put forward the hypothesis that the widespread deacetylation of lysine sites in non-histone proteins identified in HCC tumor tissues was possibly associated with the function of SIRT1-5. Then we detected expression levels of SIRT1-5 in four pairs of tumor and normal liver tissues from HCC patients. Results showed that SIRT1, 2, and 4 were up-regulated in tumor tissues while SIRT3 and 5 displayed decreased expression levels (Supplementary Figure S4). Furthermore, we collected another twenty pairs of tumor and normal liver tissues from HCC male patients. The age of patients ranged from 35 to 67, and about 57% of them were HBV positive. We detected the expression levels of SIRT1, 2, and 4 by western blot and results in Figure 7C showed that the frequencies of over-expression of SIRT1, 2, and 4 in tumor tissues were 85%, 80%, and 72%, respectively. Compared to normal liver tissues, SIRT1 (*p* = 0.002), SIRT2 (*p* = 0.01), and SIRT4 (*p* = 0.045) were significantly up-regulated in tumor tissues. These results indicated that up-regulation of SIRT 1, 2, and 4 in HCC tumor tissues possibly accounted for the deacetylation of non-histone proteins located in mitochondria and cytoplasm. Besides, it provided sirtuins as promising biomarker and therapeutic target candidates for HCC diagnosis and cure.

## Discussion

In recent years, with the development of LC-MS/MS technique and its application in proteomics study, an increasing number of lysine acetylation sites in non-histone proteins were identified in liver tissues (Zhao et al., 2010; Lundby et al., 2012). More and more evidence showed that lysine acetylation played an important role in metabolic function and signal transduction during HCC development (Ding et al., 2017; Guo et al., 2018). To better understand the function of lysine acetylation during HCC development, we performed lysine acetylome study and identified a large number of lysine sites in proteins with differential acetylation levels in HCC tumor and normal liver tissues.

In this study, the differentially acetylated proteins mainly consisted of non-histone proteins that were normally located in mitochondria and cytoplasm, where metabolic enzymes accounted for a large amount. Lysine acetylation was vital to an enzyme’s function, and disordered lysine acetylation level lead to enzyme dysfunction and metabolic syndrome (Lin et al., 2016). Metabolic rewiring including Warburg effect, enhanced fatty acid metabolism and glutamine consumption was a common feature of HCC, which provided energy for tumor growth and metastasis (Kitamura et al., 2011; Mashek et al., 2015; Infantino et al., 2019; Daye and Wellen, 2012). Consistently, the GO and KEGG pathway analysis showed that differentially modified proteins mainly participated in metabolic processes. For example, pivotal enzymes participating in glycolysis, glutaminolysis, and fatty acid oxidation processes such as PGAM2, GLUD1, HADHA, and so on, displayed aberrant acetylation levels. Besides, some of them contained more than one lysine sites with different acetylation levels in HCC tumor and normal liver tissues. Lysine acetylation modification was an important PTM that was associated with active sites and advanced structure formation. These findings provided us a new way for mechanism study of metabolic enzymes during HCC development. Besides, the differentially acetylated enzymes could serve as potential biomarkers for clinical HCC diagnosis at early stage.

In addition, we found that almost all of the proteins involved in oxidative stress showed decreased acetylation levels in HCC tumor tissues. Moreover, as the main manners for degradation and refolding of damaged and denatured proteins, proteins from the UPS and HSPs displaying disturbed lysine acetylation levels were closely connected with oxidative stress associated proteins. Oxidative stress was induced by excessive amounts of ROS produced from metabolic processes, and the antioxidant stress system was usually damaged or deficient in tumor tissues, which were vital factors for HCC initiation and progression (Birben et al., 2012; Ma-On et al., 2017). These finding not only proved the involvement of metabolic disorder and oxidative stress in promoting HCC development, but also implied the participation of acetylated proteins in ROS production and elimination processes, which might be a pivotal way for oxidative stress regulation in HCC tumor tissues.

Taken together, the lysine acetylome study provided a large amount of non-histone proteins with decreased acetylation levels in tumor tissues. Meanwhile, we found that the expression levels of SIRT 1, 2, and 4 increased in tumor tissues. Among HDACs, SIRT1, 2, and 4 were mainly located in mitochondria and cytoplasm, and they were closely related to metabolic process and oxidative stress during cancer development (Min et al., 2018; Watanabe et al., 2018; Lee et al., 2018). Besides histones, increasing numbers of non-histone proteins were identified as substrates of sirutins, which included several metabolic enzymes identified in this study (Wang L.T. et al., 2020; Xu et al., 2014). Therefore, up-regulation of SIRT 1, 2, and 4 in tumor tissues was probably responsible for the widespread deacetylation of non-histone proteins. Furthermore, researches showed that over-expression of HDACs was closely associated with cancer development, some of which were potential predictors for diagnosis and prognosis of HCC (Quint et al., 2011). HDACs inhibitors could inhibit HCC cells growth in various states including apoptosis, cell cycle arrest, and inhibition of cell migration/invasion, which made HDACs as hopeful therapeutic targets for HCC treatment (Freese et al., 2019). Therefore, over-expression of SIRT1, 2, and 4 in tumor tissues provided new promising biomarkers and drug targets for clinical HCC treatment.

In conclusion, our findings served as an important resource for the functional study of acetylated proteins in HCC development. It made insights into the association of sirtuins with widespread deacetylation of non-histone proteins in HCC tumor tissues, and provided promising diagnostic biomarkers and therapeutic targets for clinical HCC treatment.

## Data Availability Statement

The mass spectrometry proteomics data have been deposited to the ProteomeXchange Consortium (http://www.ebi.ac.uk/pride/archive/) via the PRIDE partner repository with the dataset identifier PXD014915.

## Ethics Statement

The studies involving human participants were reviewed and approved by Research Ethics Committee of Zhengzhou University. The patients/participants provided their written informed consent to participate in this study.

## Author Contributions

ML, BZ, and YL performed information verification of patients and samples collection. HC and JY contributed to introduction and discussion of this study. QZ, ZZ, JL, and FX contributed to bioinformatics analysis. QZ and ZZ performed western blot analysis. QZ wrote the manuscript. JZ supervised all phases of this study and proofread the manuscript. All authors have read and agreed to the published version of the manuscript.

## Conflict of Interest

The authors declare that the research was conducted in the absence of any commercial or financial relationships that could be construed as a potential conflict of interest.

## Footnotes

1 https://www.ebi.ac.uk/GOA/

2 http://www.genome.jp/kegg/

3 http://www.ebi.ac.uk/interpro/

4 https://string-db.org/cgi/input.pl
