# Supplementary material for: Lysine Acetylome Study of Human Hepatocellular Carcinoma Tissues for Biomarkers and Therapeutic Targets Discovery
Source: Front Genet. 2020 Sep 17;11:572663. doi: 10.3389/fgene.2020.572663 (PMC7527632; doi:10.3389/fgene.2020.572663)
Supplement: Supplementary file 1 [file Presentation_1.pdf]

Supplementary material

Table 1. Differentially acetylated lysine sites and proteins identified in HCC tumor and normal liver tissues.

Protein accession	position	Amino site	Protein description	Gene name	Modified sequence
P80404	318	K	4-aminobutyrate aminotransferase, mitochondrial	ABAT	K(1)HGCAFLVDEVQTGGGCTGK
P09110	237	K	3-ketoacyl-CoA thiolase, peroxisomal	ACAA1	GCFQAEIVPVTTTVHDDK(0.036)GTK(0.964)R
P42765	137	K	3-ketoacyl-CoA thiolase, mitochondrial	ACAA2	FGTK(1)LGSDIK
P16219	338	K	Short-chain specific acyl-CoA dehydrogenase, mitochondrial	ACADS	DNK(0.98)K(0.02)PFIK
P45954	361	K	Short/branched chain specific acyl-CoA dehydrogenase, mitochondrial	ACADSB	LLEAGK(1)PFIK
P49748	635	K	Very long-chain specific acyl-CoA dehydrogenase, mitochondrial	ACADVL	NFK(1)SISK
P49748	276	K	Very long-chain specific acyl-CoA dehydrogenase, mitochondrial	ACADVL	TPVTDPATGAVK(1)EK
P49748	298	K	Very long-chain specific acyl-CoA dehydrogenase, mitochondrial	ACADVL	GFGGITHGPPEK(1)K
P24752	257	K	Acetyl-CoA acetyltransferase, mitochondrial	ACAT1	GQPDVVVKEDEEYK(1)R
Q8N9L9	380	K	Acyl-coenzyme A thioesterase 4	ACOT4	LLNK(1)HVIWGGEPR
Q9Y305	157	K	Acyl-coenzyme A thioesterase 9, mitochondrial	ACOT9	K(1)SLSPEQDIK
Q15067	500	K	Peroxisomal acyl-coenzyme A oxidase 1	ACOX1	LVEIAAK(1)NLQK
Q68CK6	532	K	Acyl-coenzyme A synthetase ACSM2B, mitochondrial	ACSM2B	ELQQHVK(1)SVTAPYK
Q68CK6	77	K	Acyl-coenzyme A synthetase ACSM2B, mitochondrial	ACSM2B	GK(1)ELMWNFR

Q68CK6	195	K	Acyl-coenzyme A synthetase ACSM2B, mitochondrial	ACSM2B	SCDGWLNFK(1)K
Q9NR19	418	K	Acetyl-coenzyme A synthetase, cytoplasmic	ACSS2	LLMK(1)FGDEPVTK
O43707	437	K	Alpha-actinin-4	ACTN4	EAMLK(1)HR
P00326	105	K	Alcohol dehydrogenase 1C	ADH1C	ICK(1)NPESNYCLK
P00326	227	K	Alcohol dehydrogenase 1C	ADH1C	IIAVDINK(1)DK
P00326	339	K	Alcohol dehydrogenase 1C	ADH1C	LVADFMAK(1)K
P00326	114	K	Alcohol dehydrogenase 1C	ADH1C	NPESNYCLK(1)NDLGNPR
P11766	226	K	Alcohol dehydrogenase class-3	ADH5	IIGVDINK(1)DK
Q8IWW8	69	K	Hydroxyacid-oxoacid transhydrogenase, mitochondrial	ADHFE1	EVGMDLK(1)NMGAK
P35573	1302	K	Glycogen debranching enzyme	AGL	WLLELSK(1)K
P35573	1303	K	Glycogen debranching enzyme	AGL	K(1)NIFPYHEVTVK
Q9BSE5	217	K	Agmatinase, mitochondrial	AGMAT	CVDEGLLDCK(1)R
Q9BSE5	175	K	Agmatinase, mitochondrial	AGMAT	K(1)HGPVGLLHVDAHTDTTDK
O95831	593	K	Apoptosis-inducing factor 1, mitochondrial	AIFM1	IIK(1)DGEQHEDLNEVAK
Q9UIJ7	165	K	GTP:AMP phosphotransferase AK3, mitochondrial	AK3	EDDK(1)PETVIK
P27144	179	K	Adenylate kinase 4, mitochondrial	AK4	DVAK(1)PVIELYK
P27144	186	K	Adenylate kinase 4, mitochondrial	AK4	DVAKPVIELYK(1)SR
Q04828	207	K	Aldo-keto reductase family 1 member C1	AKR1C1	LLDFCK(1)SK
P00352	362	K	Retinal dehydrogenase 1	ALDH1A1	ILDLIESGK(1)K
P00352	412	K	Retinal dehydrogenase 1	ALDH1A1	FK(1)SLDDVIK
O75891	660	K	Cytosolic 10-formyltetrahydrofolate dehydrogenase	ALDH1L1	HIMK(1)SCAISNVK
P05091	368	K	Aldehyde dehydrogenase, mitochondrial	ALDH2	TEQGPQVDETQFK(1)K
P30038	93	K	Delta-1-pyrroline-5-carboxylate dehydrogenase, mitochondrial	ALDH4A1	VAK(1)FCYADK

P30038	402	K	Delta-1-pyrroline-5-carboxylate dehydrogenase, mitochondrial	ALDH4A1	K(1)WLEHAR
P30038	119	K	Delta-1-pyrroline-5-carboxylate dehydrogenase, mitochondrial	ALDH4A1	K(0.002)EWDLK(0.998)PIADR
P51649	365	K	Succinate-semialdehyde dehydrogenase, mitochondrial	ALDH5A1	AFAEAMK(1)K
Q02252	47	K	Methylmalonate-semialdehyde dehydrogenase [acylating], mitochondrial	ALDH6A1	LFIGGK(1)FVESK
P07355	227	K	Annexin A2	ANXA2	SVPHLQK(1)VFDR
P09525	246	K	Annexin A4	ANXA4	NK(1)SAYFAEK
P08133	81	K	Annexin A6	ANXA6	YELTGK(1)FER
P08133	579	K	Annexin A6	ANXA6	MTNYDVEHTIK(1)K
O15144	275	K	Actin-related protein 2/3 complex subunit 2	ARPC2	TSDFLK(1)VLNR
P00966	340	K	Argininosuccinate synthase	ASS1	HCIAK(1)SQER
P25705	305	K	ATP synthase subunit alpha, mitochondrial	ATP5A1	DNGK(1)HALIIYDDLSK
P36542	39	K	ATP synthase subunit gamma, mitochondrial	ATP5C1	SIK(1)NIQK
Q5VTU8	28	K	ATP synthase subunit epsilon-like protein, mitochondrial	ATP5EP2	DALK(1)TEFK
P24539	162	K	ATP synthase F(0) complex subunit B1, mitochondrial	ATP5F1	SQQALVQK(1)R
O75947	72	K	ATP synthase subunit d, mitochondrial	ATP5H	AGLVDDFEK(1)K
O75947	117	K	ATP synthase subunit d, mitochondrial	ATP5H	IVEYEK(1)EMEK
P48047	60	K	ATP synthase subunit O, mitochondrial	ATP5O	LEQVEK(1)ELLR
P48047	54	K	ATP synthase subunit O, mitochondrial	ATP5O	QNK(1)LEQVEK
Q13825	100	K	Methylglutaconyl-CoA hydratase, mitochondrial	AUH	AYGK(1)NSLSK

Q9BUT1	61	K	3-hydroxybutyrate dehydrogenase type 2	BDH2	VLDVTK(1)K
Q86WA6	257	K	Valacyclovir hydrolase	BPHL	FHADFIHK(1)HVK
Q86WA6	126	K	Valacyclovir hydrolase	BPHL	DAVDLMK(1)ALK
Q86WA6	208	K	Valacyclovir hydrolase	BPHL	TCEK(1)WVDGIR
Q86WA6	217	K	Valacyclovir hydrolase	BPHL	QFK(1)HLPDGNICR
P04040	476	K	Catalase	CAT	DAQIFIQK(1)K
P04040	243	K	Catalase	CAT	TDQGIK(1)NLSVEDAAR
Q8N4T8	195	K	Carbonyl reductase family member 4	CBR4	DLKEEHLK(1)K
P40227	199	K	T-complex protein 1 subunit zeta	CCT6A	HK(1)SETDTSLIR
P50990	400	K	T-complex protein 1 subunit theta	CCT8	AVDDGVNTFK(1)VLTR
Q9NZ45	104	K	CDGSH iron-sulfur domain-containing protein 1	CISD1	HNEETGDNVGPLIIK(1)K
Q9NZ45	79	K	CDGSH iron-sulfur domain-containing protein 1	CISD1	K(1)FPFCDGAHTK
Q8N0X4	57	K	Citrate lyase subunit beta-like protein, mitochondrial	CLYBL	AVLYVPGNDEK(1)K
P09543	178	K	2',3'-cyclic-nucleotide 3'-phosphodiesterase	CNP	LK(1)PGLEK
P10606	56	K	Cytochrome c oxidase subunit 5B, mitochondrial	COX5B	EIMLAAK(1)K
P10606	86	K	Cytochrome c oxidase subunit 5B, mitochondrial	COX5B	EDPNLVPSISNK(1)R
P31327	919	K	Carbamoyl-phosphate synthase [ammonia], mitochondrial	CPS1	QISK(1)CLGLTEAQTR
P31327	527	K	Carbamoyl-phosphate synthase [ammonia], mitochondrial	CPS1	RGVLK(1)EYGVK
P31327	1150	K	Carbamoyl-phosphate synthase [ammonia], mitochondrial	CPS1	K(1)FLEEATR
P43155	268	K	Carnitine O-acetyltransferase	CRAT	AYNTLIK(1)DK
Q08257	120	K	Quinone oxidoreductase	CRYZ	LPEK(1)LDFK
P04080	91	K	Cystatin-B	CSTB	AK(1)HDELTYF
P99999	40	K	Cytochrome c	CYCS	K(1)TGQAPGYSYTAANK
P07108	55	K	Acyl-CoA-binding protein	DBI	AK(1)WDAWNELK
P07108	77	K	Acyl-CoA-binding protein	DBI	AYINK(1)VEELK

Q96C86	138	K	m7GpppX diphosphatase	DCPS	TTVVYPATEK(1)HLQK
P30046	21	K	D-dopachrome decarboxylase	DDT	VPAGLEK(1)R
Q96HY7	827	K	Probable 2-oxoglutarate dehydrogenase E1 component DHKTD1, mitochondrial	DHTKD1	K(1)HDFAIIR
P10515	466	K	Dihydrolipoyllysine-residue acetyltransferase component of pyruvate dehydrogenase complex, mitochondrial	DLAT	ELNK(1)ILEGR
P09622	166	K	Dihydrolipoyl dehydrogenase, mitochondrial	DLD	NQVTATK(1)ADGGTQVIDTK
P09622	277	K	Dihydrolipoyl dehydrogenase, mitochondrial	DLD	LNTK(1)VTGATK
P09622	159	K	Dihydrolipoyl dehydrogenase, mitochondrial	DLD	ITGK(1)NQVTATK
P36957	267	K	Dihydrolipoyllysine-residue succinyltransferase component of 2-oxoglutarate dehydrogenase complex, mitochondrial	DLST	HK(1)EAFLK(1)K
Q9UI17	360	K	Dimethylglycine dehydrogenase, mitochondrial	DMGDH	AAMEMVPVLK(1)K
Q9ULA0	23	K	Aspartyl aminopeptidase	DNPEP	ELLK(1)FVNR
Q96N67	1962	K	Dedicator of cytokinesis protein 7	DOCK7	AHGELHEQFK(1)R
Q13011	95	K	Delta(3,5)-Delta(2,4)-dienoyl-CoA isomerase, mitochondrial	ECH1	EMVECFNK(1)ISR
Q9NTX5	108	K	Ethylmalonyl-CoA decarboxylase	ECHDC1	GAK(1)NTFSSGSDLNAVK
Q96DC8	65	K	Enoyl-CoA hydratase domain-containing protein 3, mitochondrial	ECHDC3	NIVLSNPK(1)K
P30084	118	K	Enoyl-CoA hydratase, mitochondrial	ECHS1	FLK(1)HWDHLTQVK

P30084	261	K	Enoyl-CoA hydratase, mitochondrial	ECHS1	K(1)LFYSTFATDDRK
O75521	161	K	Enoyl-CoA delta isomerase 2, mitochondrial	ECI2	K(1)NAINTEMYHEIMR
Q5VTE0	179	K	Putative elongation factor 1-alpha-like 3	EEF1A1P5	EVSTYIK(1)K
P26641	434	K	Elongation factor 1-gamma	EEF1G	AFNQGK(1)IFK
Q08426	605	K	Peroxisomal bifunctional enzyme	EHHADH	K(1)THHIEPR
Q08426	577	K	Peroxisomal bifunctional enzyme	EHHADH	TGK(1)GWYQYDKPLGR
P60228	275	K	Eukaryotic translation initiation factor 3 subunit E	EIF3E	QVLK(1)DLVK
Q09472	1550	K	Histone acetyltransferase p300	EP300	EENTSNESTDVTK(1)GDSK(1)NAK(1)K(1)K
P07099	447	K	Epoxide hydrolase 1	EPHX1	K(1)FLSVLER
P07099	286	K	Epoxide hydrolase 1	EPHX1	DVELLYPVK(1)EK
P34913	94	K	Bifunctional epoxide hydrolase 2	EPHX2	EIFDK(1)AISAR
P13804	62	K	Electron transfer flavoprotein subunit alpha, mitochondrial	ETFA	CDK(1)VAQDLCK
P13804	226	K	Electron transfer flavoprotein subunit alpha, mitochondrial	ETFA	GLK(1)SGENFK
O95571	172	K	Persulfide dioxygenase ETHE1, mitochondrial	ETHE1	TDFQQGCAK(1)TLYHSVHEK
P49327	528	K	Fatty acid synthase	FASN	SDEAVK(1)PFGLK
P07954	223	K	Fumarate hydratase, mitochondrial	FH	SK(1)EFAQIIK
O95954	427	K	Formimidoyltransferase-cyclodeaminase	FTCD	LPK(1)NTPEEKDR
P04406	117	K	Glyceraldehyde-3-phosphate dehydrogenase	GAPDH	AGAHLQGGAK(1)R
P04406	194	K	Glyceraldehyde-3-phosphate dehydrogenase	GAPDH	TVDGPSGK(1)LWR
P50440	385	K	Glycine amidinotransferase, mitochondrial	GATM	MFEK(1)LGITTIK
P50395	269	K	Rab GDP dissociation inhibitor beta	GDI2	VIGVK(1)SEGEIAR

P23378	423	K	Glycine dehydrogenase (decarboxylating), mitochondrial	GLDC	VHNATLILSEGLK(1)R
P00367	457	K	Glutamate dehydrogenase 1, mitochondrial	GLUD1	LTFK(1)YER
P00367	191	K	Glutamate dehydrogenase 1, mitochondrial	GLUD1	INPK(1)NYTDNELEK
P00367	390	K	Glutamate dehydrogenase 1, mitochondrial	GLUD1	QLTK(1)SNAPR
P00367	527	K	Glutamate dehydrogenase 1, mitochondrial	GLUD1	TAMK(1)YNLGLDLR
P00367	480	K	Glutamate dehydrogenase 1, mitochondrial	GLUD1	FGK(1)HGGTIPIVPTAEFQDR
Q6IB77	20	K	Glycine N-acyltransferase	GLYAT	K(1)SLPASLK
P00505	159	K	Aspartate aminotransferase, mitochondrial	GOT2	DVFLPK(1)PTWGNHTPIFR
P00505	73	K	Aspartate aminotransferase, mitochondrial	GOT2	DDNGK(1)PYVLPSVR
Q9UBQ7	327	K	Glyoxylate reductase/hydroxypyruvate reductase	GRHPR	GEPMPSELK(1)L
P08263	138	K	Glutathione S-transferase A1	GSTA1	YFPAFEK(1)VLK
P08263	84	K	Glutathione S-transferase A1	GSTA1	YNLYGK(1)DIK
P08263	36	K	Glutathione S-transferase A1	GSTA1	FIK(1)SAEDLDK
P08263	141	K	Glutathione S-transferase A1	GSTA1	VLK(1)SHGQDYLVGNK
P08263	43	K	Glutathione S-transferase A1	GSTA1	SAEDLDK(1)LR
Q9Y2Q3	169	K	Glutathione S-transferase kappa 1	GSTK1	VK(1)NQLK(1)ETTEAACR
P78417	160	K	Glutathione S-transferase omega-1	GSTO1	LEEVLTNK(1)K
P78417	59	K	Glutathione S-transferase omega-1	GSTO1	NK(1)PEWFFK
O75367	304	K	Core histone macro-H2A.1	H2AFY	NCLALADDK(1)K
Q16836	132	K	Hydroxyacyl-coenzyme A dehydrogenase, mitochondrial	HADH	VKNELFK(1)R

Q16836	241	K	Hydroxyacyl-coenzyme A dehydrogenase, mitochondrial	HADH	GDASK(1)EDIDTAMK
P40939	759	K	Trifunctional enzyme subunit alpha, mitochondrial	HADHA	QFTPCQLLADHANSPNK(1)K
P40939	60	K	Trifunctional enzyme subunit alpha, mitochondrial	HADHA	INSPNSK(1)VNTLSK
P40939	129	K	Trifunctional enzyme subunit alpha, mitochondrial	HADHA	IVEK(1)LEK
P40939	605	K	Trifunctional enzyme subunit alpha, mitochondrial	HADHA	HVAEDLGK(1)VFGER
P40939	569	K	Trifunctional enzyme subunit alpha, mitochondrial	HADHA	ILQEGVDPK(1)K
P55084	181	K	Trifunctional enzyme subunit beta, mitochondrial	HADHB	K(1)LMLDLNK
P55084	188	K	Trifunctional enzyme subunit beta, mitochondrial	HADHB	LMLDLNK(1)AK
P55084	253	K	Trifunctional enzyme subunit beta, mitochondrial	HADHB	SHSLAK(1)K
P55084	190	K	Trifunctional enzyme subunit beta, mitochondrial	HADHB	AK(1)SMGQR
Q93099	98	K	Homogentisate 1,2-dioxygenase	HGD	WK(1)PFEIPK
Q6NVY1	360	K	3-hydroxyisobutyryl-CoA hydrolase, mitochondrial	HIBCH	WK(1)PADLK
P49773	30	K	Histidine triad nucleotide-binding protein 1	HINT1	EIPAK(1)IIFEDDR
P10412	17	K	Histone H1.4	HIST1H1E	SETAPAAPAAPAPAEK(1)TPVK
P35914	48	K	Hydroxymethylglutaryl-CoA lyase, mitochondrial	HMGCL	DGLQNEK(1)NIVSTPVK
P54868	354	K	Hydroxymethylglutaryl-CoA synthase, mitochondrial	HMGCS2	DLDK(1)ALLK

P54868	358	K	Hydroxymethylglutaryl-CoA synthase, mitochondrial	HMGCS2	ALLK(1)ASQDMFDKK
Q00839	352	K	Heterogeneous nuclear ribonucleoprotein U	HNRNPU	HLYTK(1)DIDIHEVR
P51659	644	K	Peroxisomal multifunctional enzyme type 2	HSD17B4	DIGPEVVK(1)K
P51659	84	K	Peroxisomal multifunctional enzyme type 2	HSD17B4	VVK(1)TALDAFGR
P51659	707	K	Peroxisomal multifunctional enzyme type 2	HSD17B4	LDPQK(1)AFFSGR
P51659	184	K	Peroxisomal multifunctional enzyme type 2	HSD17B4	K(1)SNIHCNTIAPNAGSR
P11142	246	K	Heat shock cognate 71 kDa protein	HSPA8	MVNHFIAEFK(1)R
P38646	612	K	Stress-70 protein, mitochondrial	HSPA9	LK(1)EEISK
P38646	138	K	Stress-70 protein, mitochondrial	HSPA9	DIK(1)NVPFK
P38646	135	K	Stress-70 protein, mitochondrial	HSPA9	RYDDPEVQK(1)DIK
P38646	288	K	Stress-70 protein, mitochondrial	HSPA9	HIVK(1)EFK
P10809	125	K	60 kDa heat shock protein, mitochondrial	HSPD1	SIAK(1)EGFEK
P10809	91	K	60 kDa heat shock protein, mitochondrial	HSPD1	YK(1)NIGAK
Q9NSE4	725	K	Isoleucine--tRNA ligase, mitochondrial	IARS2	DDISK(1)LR
O75874	236	K	Isocitrate dehydrogenase [NADP] cytoplasmic	IDH1	QYK(1)SQFEAQK
O75874	65	K	Isocitrate dehydrogenase [NADP] cytoplasmic	IDH1	DAAEAIK(1)K
O75874	81	K	Isocitrate dehydrogenase [NADP] cytoplasmic	IDH1	CATITPDEK(1)R
P48735	275	K	Isocitrate dehydrogenase [NADP], mitochondrial	IDH2	HYK(1)TDFDK
P48735	256	K	Isocitrate dehydrogenase [NADP], mitochondrial	IDH2	NTILK(1)AYDGR

P26440	55	K	Isovaleryl-CoA dehydrogenase, mitochondrial	IVD	QTMAK(1)FLQEHLAPK
P26440	75	K	Isovaleryl-CoA dehydrogenase, mitochondrial	IVD	SNEFK(1)NLR
P26440	238	K	Isovaleryl-CoA dehydrogenase, mitochondrial	IVD	GMPGFSTSK(1)K
P00338	318	K	L-lactate dehydrogenase A chain	LDHA	K(1)SADTLWGIQK
P02545	470	K	Prelamin-A/C	LMNA	SNEDQSMGNWQIK(1)R
P02545	180	K	Prelamin-A/C	LMNA	LEAALGEAK(1)K
P20700	532	K	Lamin-B1	LMNB1	VILK(1)NSQGEEVAQR
Q03252	195	K	Lamin-B2	LMNB2	AEDGHAVAK(1)K
P36776	917	K	Lon protease homolog, mitochondrial	LONP1	AGVTCIVLPAENK(1)K
P40925	118	K	Malate dehydrogenase, cytoplasmic	MDH1	SQGAALDK(1)YAK
P40925	110	K	Malate dehydrogenase, cytoplasmic	MDH1	IFK(1)SQGAALDK
P40926	301	K	Malate dehydrogenase, mitochondrial	MDH2	GIEK(1)NLGIGK
P40926	157	K	Malate dehydrogenase, mitochondrial	MDH2	K(1)HGVYNPNK
Q9BV79	267	K	Trans-2-enoyl-CoA reductase, mitochondrial	MECR	LALNCVGGK(1)SSTELLR
Q9NYK5	72	K	39S ribosomal protein L39, mitochondrial	MRPL39	HVGK(1)TDPGTVFVMNK
P26038	258	K	Moesin	MSN	FVIK(1)PIDK
P26038	253	K	Moesin	MSN	NISFNDK(1)K
P26038	79	K	Moesin	MSN	ESPLLFK(1)FR
P11586	21	K	C-1-tetrahydrofolate synthase, cytoplasmic	MTHFD1	LK(1)NQVTQLK
P11586	364	K	C-1-tetrahydrofolate synthase, cytoplasmic	MTHFD1	LK(1)HRPDGK
P22033	340	K	Methylmalonyl-CoA mutase, mitochondrial	MUT	MFQPK(1)NSK
P35579	1370	K	Myosin-9	MYH9	QIATLHAQVADMK(1)K
P19338	646	K	Nucleolin	NCL	VTLDWAK(1)PK
O43676	34	K	NADH dehydrogenase [ubiquinone] 1 beta subcomplex subunit 3	NDUFB3	IEGTPLETIQK(1)K

O95139	66	K	NADH dehydrogenase [ubiquinone] 1 beta subcomplex subunit 6	NDUFB6	MVHGVYK(1)K
Q9UFN0	166	K	Protein NipSnap homolog 3A	NIPSNAP3A	PGGPALWGDAFK(1)R
P40261	39	K	Nicotinamide N-methyltransferase	NNMT	HSAESQILK(1)HLLK
Q13423	70	K	NAD(P) transhydrogenase, mitochondrial	NNT	EIFQNEK(1)R
Q13423	171	K	NAD(P) transhydrogenase, mitochondrial	NNT	K(1)TTVLAMDQVPR
Q15738	126	K	Sterol-4-alpha-carboxylate 3-dehydrogenase, decarboxylating	NSDHL	VNYIGTK(1)NVIETCK
Q9NTK5	248	K	Obg-like ATPase 1	OLA1	IK(1)EWVDK
P00480	46	K	Ornithine carbamoyltransferase, mitochondrial	OTC	DLLTLK(1)NFTGEEIK
P00480	307	K	Ornithine carbamoyltransferase, mitochondrial	OTC	K(1)PEEVDDEVFYSPR
P00480	88	K	Ornithine carbamoyltransferase, mitochondrial	OTC	SLGMIFEK(1)R
P00480	144	K	Ornithine carbamoyltransferase, mitochondrial	OTC	VYK(1)QSDLDTLAK
P07237	31	K	Protein disulfide-isomerase	P4HB	K(1)SNFAEALAAHK
Q9H0N5	125	K	Pterin-4-alpha-carbinolamine dehydratase 2	PCBD2	FIEK(1)AAASV
P05165	150	K	Propionyl-CoA carboxylase alpha chain, mitochondrial	PCCA	AQAVHPGYGFLSENK(1)EFAR
P05165	65	K	Propionyl-CoA carboxylase alpha chain, mitochondrial	PCCA	TFDK(1)ILVANR
P05165	519	K	Propionyl-CoA carboxylase alpha chain, mitochondrial	PCCA	GHMLTK(1)SEK
P05166	101	K	Propionyl-CoA carboxylase beta chain, mitochondrial	PCCB	NK(1)FPGDSVVTGR

P08559	313	K	Pyruvate dehydrogenase E1 component subunit alpha, somatic form, mitochondrial	PDHA1	SK(1)SDPIMLLK
Q9BY49	291	K	Peroxisomal trans-2-enoyl-CoA reductase	PECR	GAGDLSVVK(1)K
P17858	726	K	ATP-dependent 6-phosphofructokinase, liver type	PFKL	AVAFSPVTELK(1)K
P15259	100	K	Phosphoglycerate mutase 2	PGAM2	HYGGLTGLNK(1)AETAAK
P00558	11	K	Phosphoglycerate kinase 1	PGK1	LTLDK(1)LDVK
P00558	267	K	Phosphoglycerate kinase 1	PGK1	IVK(1)DLMSK
P36871	457	K	Phosphoglucomutase-1	PGM1	SFVGK(1)QFSANDK
P30613	321	K	Pyruvate kinase PKLR	PKLR	IENHEGVK(1)R
O15305	149	K	Phosphomannomutase 2	PMM2	IEFYELDK(1)K
Q9H2U2	224	K	Inorganic pyrophosphatase 2, mitochondrial	PPA2	FHDIDDVK(1)K
P62937	125	K	Peptidyl-prolyl cis-trans isomerase A	PPIA	TEWLDGK(1)HVVFGK
P62937	44	K	Peptidyl-prolyl cis-trans isomerase A	PPIA	ALSTGEK(1)GFGYK
P30405	73	K	Peptidyl-prolyl cis-trans isomerase F, mitochondrial	PPIF	ADVVPK(1)TAENFR
Q06830	35	K	Peroxiredoxin-1	PRDX1	DISLSDYK(1)GK
P30048	91	K	Thioredoxin-dependent peroxide reductase, mitochondrial	PRDX3	DLSLDDFK(1)GK
P30041	63	K	Peroxiredoxin-6	PRDX6	LAPEFAK(1)R
P78527	3260	K	DNA-dependent protein kinase catalytic subunit	PRKDC	LLK(1)ELHK
O94903	125	K	Proline synthase co-transcribed bacterial homolog protein	PROSC	LADK(1)VNSSWQR
P25789	127	K	Proteasome subunit alpha type-4	PSMA4	QAYTQFGGK(1)R
P60900	181	K	Proteasome subunit alpha type-6	PSMA6	QTESTSFLEK(1)K
Q99460	720	K	26S proteasome non-ATPase regulatory subunit 1	PSMD1	VINDK(1)HDDVMAK
Q14914	75	K	Prostaglandin reductase 1	PTGR1	VVESK(1)NVALPK

P06737	29	K	Glycogen phosphorylase, liver form	PYGL	GIVGVENVAELK(1)K
P06737	724	K	Glycogen phosphorylase, liver form	PYGL	IDDVAALDK(1)K
P06737	78	K	Glycogen phosphorylase, liver form	PYGL	TQQHYYDK(1)CPK
P47897	628	K	Glutamine--tRNA ligase	QARS	TDFKEEPEPGFK(1)R
P09417	102	K	Dihydropteridine reductase	QDPR	SLFK(1)NCDLMWK
O75452	247	K	Retinol dehydrogenase 16	RDH16	FVADYK(1)K
P62906	91	K	60S ribosomal protein L10a	RPL10A	AVDIPHMDIEALK(1)K
P04843	516	K	Dolichyl-diphosphooligosaccharide--protein glycosyltransferase subunit 1	RPN1	DISTLNSGK(1)K
P22307	453	K	Non-specific lipid-transfer protein	SCP2	KLEEEGEQFVK(1)K
P31040	250	K	Succinate dehydrogenase [ubiquinone] flavoprotein subunit, mitochondrial	SDHA	AK(1)NTVVATGGYGR
P31040	547	K	Succinate dehydrogenase [ubiquinone] flavoprotein subunit, mitochondrial	SDHA	LYGDLK(1)HLK
Q13228	125	K	Selenium-binding protein 1	SELENBP1	VIEPK(1)DIHAK
P34896	416	K	Serine hydroxymethyltransferase, cytosolic	SHMT1	GLLEK(1)DFQK
P34897	269	K	Serine hydroxymethyltransferase, mitochondrial	SHMT2	VIPSPFK(1)HADIVTTTTHK
P34897	103	K	Serine hydroxymethyltransferase, mitochondrial	SHMT2	YSEGYPGK(1)R
Q9UJS0	379	K	Calcium-binding mitochondrial carrier protein Aralar2	SLC25A13	NSFDCFK(1)K
Q00325	214	K	Phosphate carrier protein, mitochondrial	SLC25A3	EEGLK(1)AFYK
Q00325	206	K	Phosphate carrier protein, mitochondrial	SLC25A3	DAAPK(1)MYKEEGLK
P05141	105	K	ADP/ATP translocase 2	SLC25A5	QIFLGGVDK(1)R

P05141	23	K	ADP/ATP translocase 2	SLC25A5	DFLAGGVAAAISK(1)TAVAPIER
P05141	166	K	ADP/ATP translocase 2	SLC25A5	IYK(1)SDGIK
P05141	268	K	ADP/ATP translocase 2	SLC25A5	DEGGK(1)AFFK
Q13485	45	K	Mothers against decapentaplegic homolog 4	SMAD4	AIESLVK(1)K
P08621	162	K	U1 small nuclear ribonucleoprotein 70 kDa	SNRNP70	DMHSAYK(1)HADGK
P55769	20	K	NHP2-like protein 1	SNU13	AYPLADAHLTK(1)K
P00441	123	K	Superoxide dismutase [Cu-Zn]	SOD1	TLVVHEK(1)ADDLGK
P04179	130	K	Superoxide dismutase [Mn], mitochondrial	SOD2	RDFGSFDK(1)FK
P04179	122	K	Superoxide dismutase [Mn], mitochondrial	SOD2	GELLEAIK(1)R
Q9Y6N5	135	K	Sulfide:quinone oxidoreductase, mitochondrial	SQRDL	VTELNPDKNCIHTDDDEK(1)ISYR
Q9UJZ1	145	K	Stomatin-like protein 2, mitochondrial	STOML2	LSLDK(1)VFR

Table 2. Acetylated proteins involved in UPS.

Protein	Protein description	Acetylation site	Cancer vs Normal
P60228	Eukaryotic translation initiation factor 3 subunit E	1	0.471
P36776	Lon protease homolog, mitochondrial	1	0.511
Q9NTK5	Obg-like ATPase 1	1	0.377
P60900	Proteasome subunit alpha type-6	1	0.463
P25789	Proteasome subunit alpha type-4	1	0.649
Q99460	26S proteasome non-ATPase regulatory subunit 1	1	0.606
P61088	Ubiquitin-conjugating enzyme E2 N	1	0.408

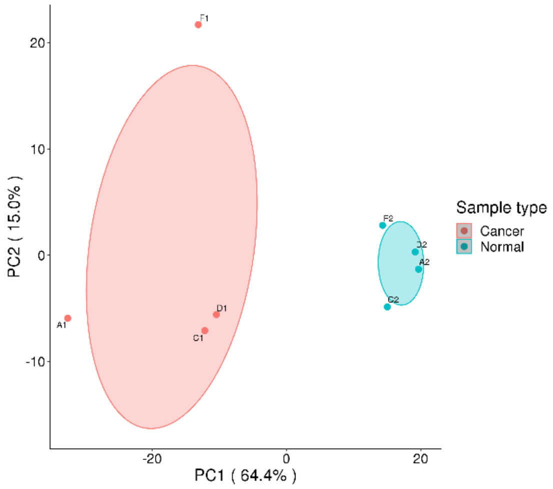

Figure 1. Principal Component Analysis (PCA) of tumor and normal liver tissues from HCC patients.

A

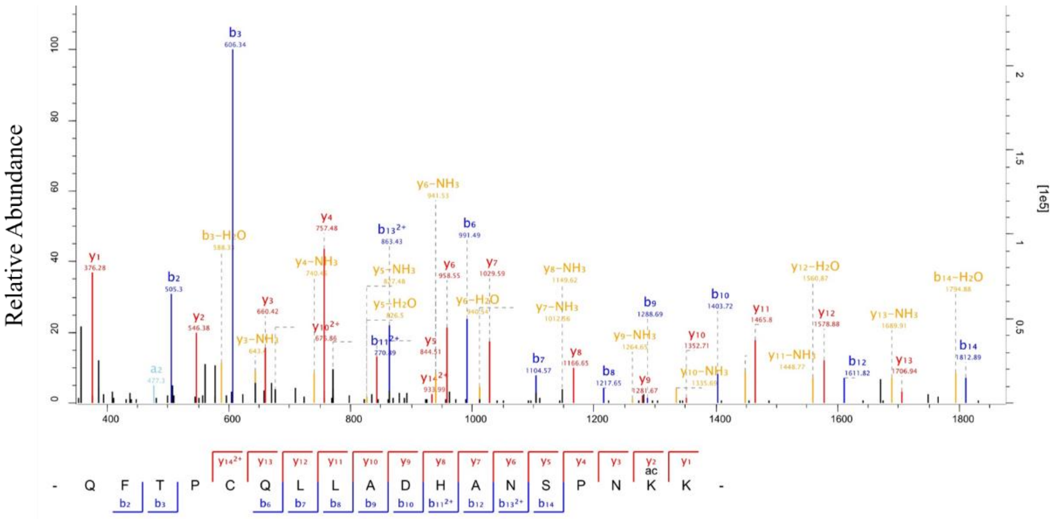

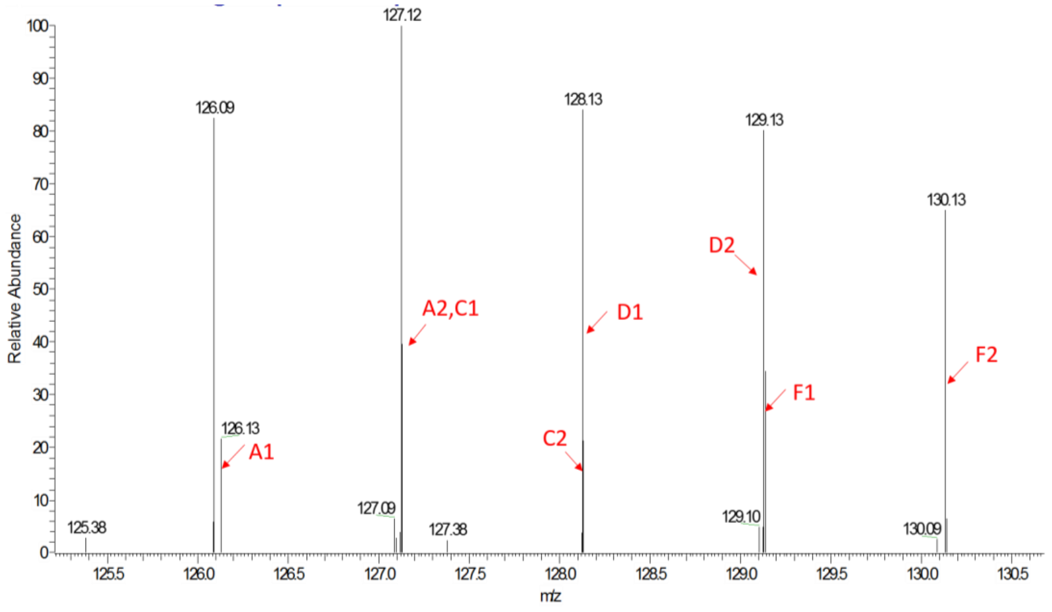

B

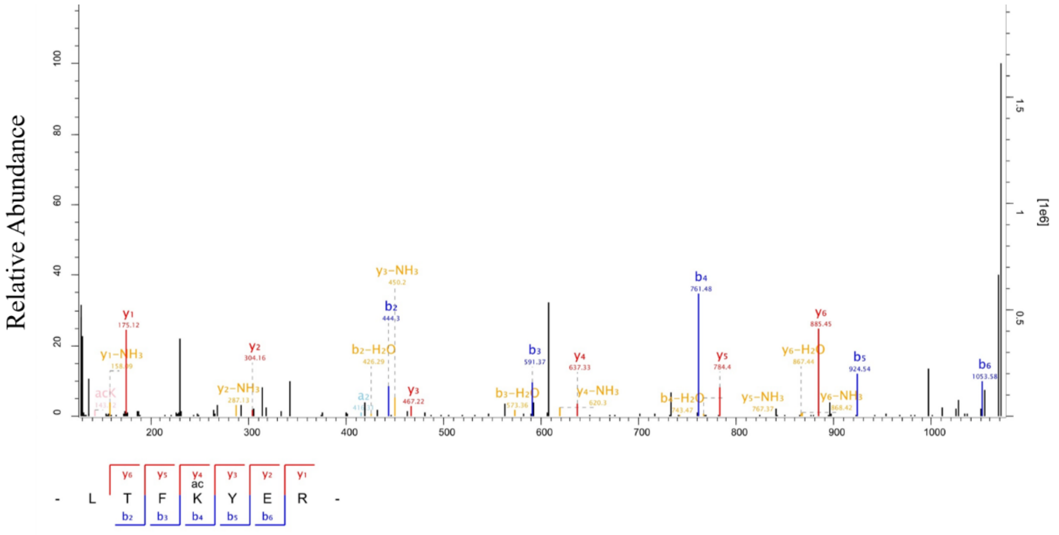
Relative Abundance
y1
175.12
y1-NH3
y2
304.16
y2-NH3
y3-NH3
450.2
b2
444.3
b2-H2O
426.29
a2
y3
467.22
b3
591.37
y4
637.33
b3-H2O
y4-NH3
b4
761.48
b4-H2O
y5
784.4
y5-NH3
y6
885.45
y6-H2O
867.44
y6-NH3
b5
924.54
b6
1053.58
[1e6]
L
T
F
K
ac
Y
E
R

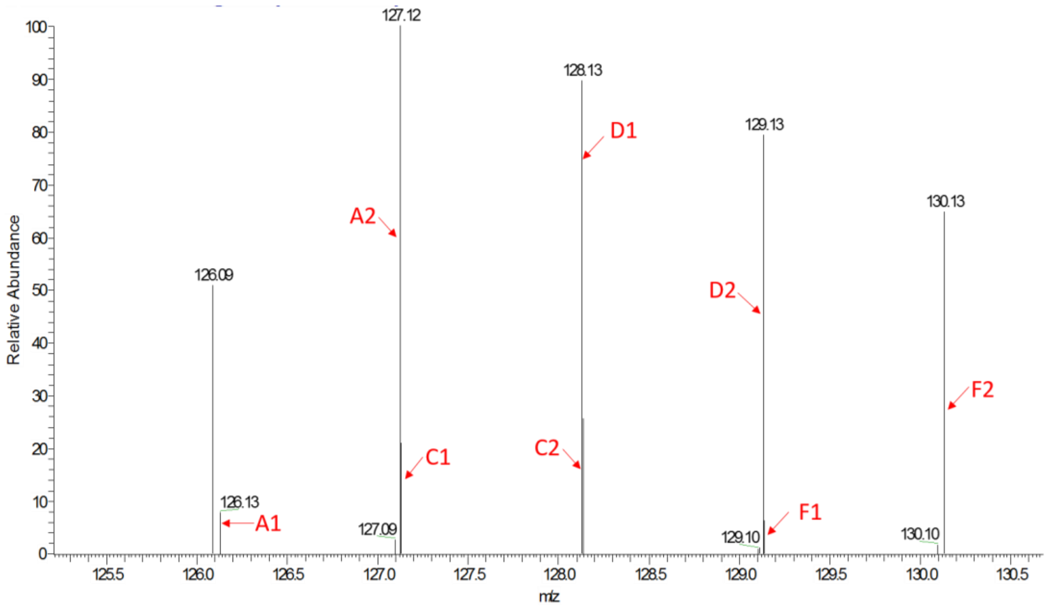
127.12
128.13
129.13
130.13
126.09
126.13
127.09
129.10
130.10
A1
A2
C1
C2
D1
D2
F1
F2
Relative Abundance
m/z

C

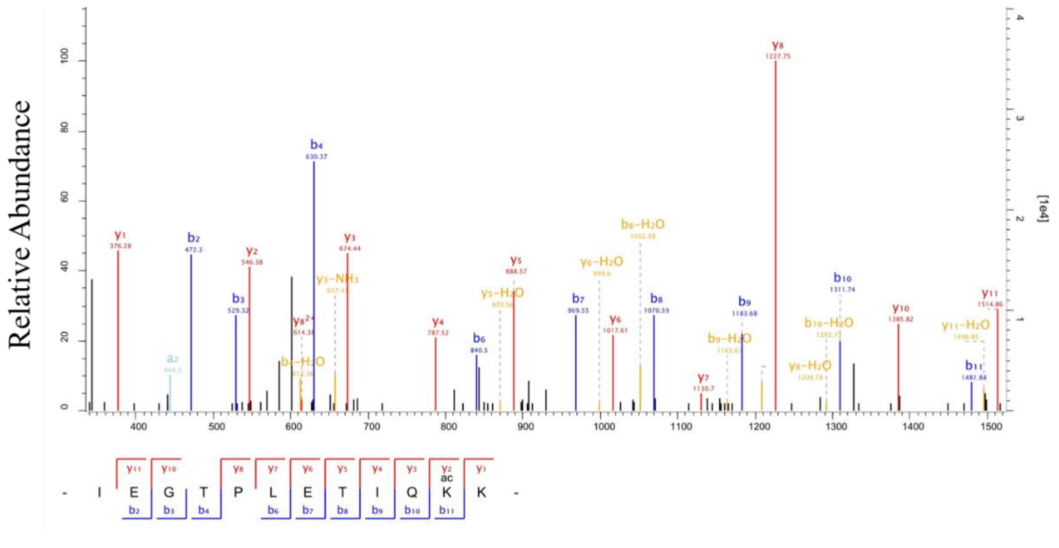
Relative Abundance
y1
376.28
b2
472.3
a2
b3
529.32
y2
546.38
b4
630.37
y3
674.44
y3-NH3
y4
787.52
b6
840.5
y5
888.57
y5-H2O
b7
969.55
y6
1017.61
y6-H2O
b8
1070.59
b8-H2O
1052.58
y7
1130.7
b9
1183.68
b9-H2O
y8
1227.75
y8-H2O
b10
1311.74
b10-H2O
y10
1385.82
b11
1481.84
y11
1514.86
y11-H2O
[1e4]
I
E
G
T
P
L
E
T
I
Q
K
ac
K

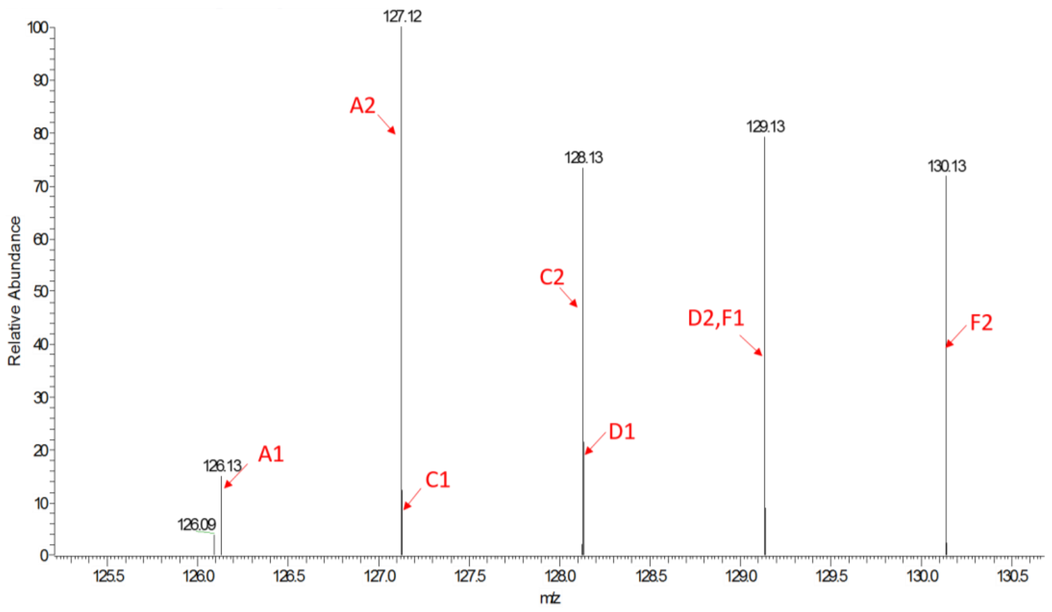

Figure 2. MS/MS spectra for HADHA (A), GLUD1 (B), NDUFB3 (C) and the relative intensities of the TMT reporter ions in the different tissue samples (A1, C1, D1 and F1 are tumor tissues; A2, C2, D2 and F2 are normal tissues) are shown below.

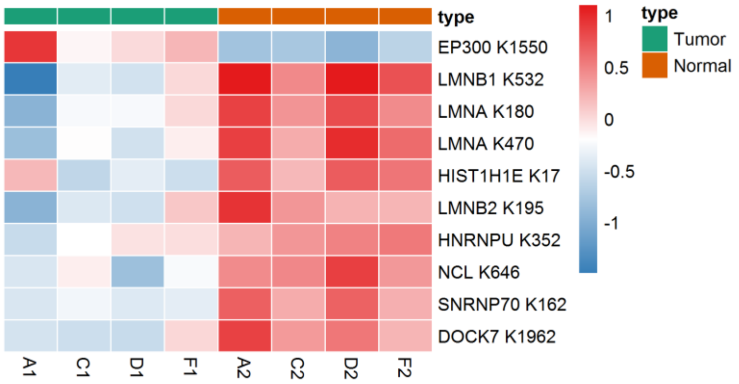

Figure 3. Acetylation levels of lysine sites in nuclear proteins displayed in the heat map.

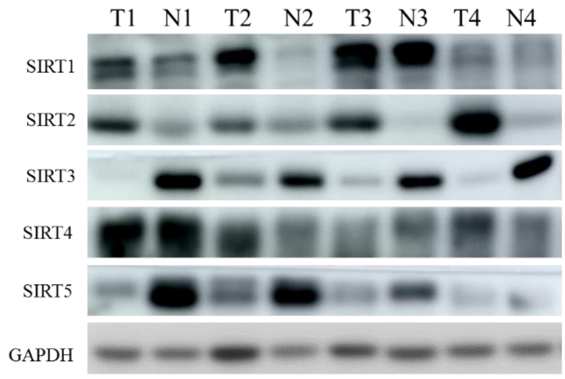

Figure 4. Western blot analysis of SIRT1-5 in four pairs of tumor and normal liver tissues from HCC patients.
